# Influence of Multiple Types of Proximity on the Degree of Visual Crowding Effects Within a Single Gap Detection Task

**DOI:** 10.1177/2041669519837263

**Published:** 2019-03-16

**Authors:** Daisuke Hayashi, Madoka Ohnishi

**Affiliations:** Department of Psychology, The University of Tokyo, Japan; Faculty of Human Informatics, Aichi Shukutoku University, Japan; Graduate School of Humanities and Sciences, Tokyo Woman’s Christian University, Japan; IdeaLab Inc., Tokyo, Japan

**Keywords:** crowding, proximity, gap detection, spatial vision, peripheral vision

## Abstract

The visual system cannot recognize an object (target) in peripheral vision when presented with neighboring similar stimuli (flanker). This object recognition disability is known as crowding. Studies have shown that various types of proximity, such as spatial distance or semantic category, affect the degree of crowding. However, thus far, these effects have mostly been studied separately. Hence, their underlying similarities and differences are still unknown. In this study, we developed a novel gap detection task and tested whether the effect of three different types of proximity in crowding (the relative position between target gap and nearest flanker edge, the flanker location compared with the target location, and the semantic category of the target) can be measured within a single task. A psychometric function analysis revealed that two of the assumed types of proximity affected the degree of crowding within a single task.

## Introduction

A fundamental function of the visual system is the recognition of objects in the environment. When a visual stimulus (target) is presented in peripheral vision together with other similar stimuli (flankers), recognizing a target is more difficult than when it is presented alone (Levi, 2008; Pelli & Tillman, 2008; Strasburger, Rentschler, & Juttner, 2011; Whitney & Levi, 2011 for reviews). This inability to recognize objects in peripheral vision is called the visual crowding effect or in short “crowding.” One attempt at understanding what underlies crowding is that the visual system inaccurately integrates features of flankers into the target in peripheral vision, resulting in confusion between flankers and the target (Pelli, Palomares, & Majaj, 2004; Pelli & Tillman, 2008; Wolford, 1975). Crowding is an important phenomenon enabling us to understand how the visual system recognizes objects.

Several studies have shown that in crowding, as a general rule, a *closer* flanker causes a *stronger* effect on target recognition. Therefore, various types of proximity influence the degree of crowding. For example, crowding weakens as target-flanker distance enlarges (Bouma, 1970). Cortical rather than physical distance has been shown to be important (Liu, Jiang, Sun, & He, 2009). Another type of spatial dependency is central-peripheral asymmetry, wherein flankers presented more peripherally than the target cause stronger crowding than those presented more centrally than the target (Bex, Dakin, & Simmers, 2003; Bouma, 1973; Chastain, 1983; Dayan & Solomon, 2010; Estes, Allmeyer, & Reder, 1976; Estes & Wolford, 1971; Haslerud & Clark, 1957; Krumhansl, 1977; Mackworth, 1965; Petrov & Popple, 2007; Petrov, Popple, & McKee, 2007; Toet & Levi, 1992; see Strasburger, 2018, for a recent review; see also Chastain, 1982; Strasburger & Malania, 2013 showing the opposite results). One possible explanation of such central-peripheral asymmetry is related to the cortical magnification factor (Motter & Simoni, 2007; Nandy & Tjan, 2012). The representation of the distance between the target and flanker in the visual cortex is smaller when the flanker is presented more peripherally than the target than when the flanker is presented more centrally than the target. Therefore, regarding cortical distance, more peripheral flankers are represented closer to the target than those more central. Furthermore, both spatial proximity and, more abstractly, the proximity in feature space also affect crowding. According to previous psychophysical studies, crowding is more evident when the target and flankers have similar properties than when they have different ones, such as luminance polarity (Chakravarthi & Cavanagh, 2007; Kooi, Toet, Tripathy, & Levi, 1994), orientation (Wilkinson, Wilson, & Ellemberg, 1997), spatial frequency (Chung, Levi, & Legge, 2001), and color (Kennedy & Whitaker, 2010; van den Berg, Roerdink, & Cornelissen, 2007). Moreover, crowding is observed to be stronger when the semantic categories of the flankers and the target are the same than when they are different. Specifically, crowding was more evident when both target and flanker were from the same category, such as faces (Farzin, Rivera, & Whitney, 2009; Louie, Bressler, & Whitney, 2007) or letters (Huckauf, Heller, & Nazir, 1999; Reuther & Chakravarthi, 2014; but see also Yu, Akau, & Chung, 2012), than when they were in different ones. In summary, crowding occurs especially when the visual system cannot appropriately dissociate the target from flankers because of their proximity in various dimensions.

Each type of proximity has been primarily studied in separate tasks or stimuli. Such approaches are important to examine each effect in detail. However, to investigate their similarities and differences, the influences of different types of proximity should be tested within a single task. In this study, we developed a novel gap detection task and tested whether it is possible to measure the effect of three different types of proximity on crowding within a single task.

## Methods

### Observers

Including the first author, 11 observers with normal or corrected-to-normal visual acuity participated in this study. All the observers except for the first author were unaware of the purpose of the experiment. They submitted written informed consent. The experiments were approved by the ethics committee of The University of Tokyo and were conducted in accordance with the Declaration of Helsinki. Constrained by a chin rest, each observer viewed visual stimuli in a dark room at a distance of 57 cm and with both eyes open.

### Apparatus

Stimuli were generated by an Apple MacPro and were displayed on a CRT monitor (Iiyama HM204DA). The luminance profile of the monitor was gamma corrected. The spatial resolution of the monitor was 1280 pixel × 960 pixel; its refresh rate was 120 Hz. The MATLAB programming environment and Psychophysics Toolbox extensions were used to generate visual stimuli (Brainard, 1997; Kleiner, Brainard, & Pelli, 2007; Pelli, 1997).

### Stimuli

Stimuli were presented on a uniform gray background (46 cd/m^2^); each stimulus was colored black (0.36 cd/m^2^). Stimuli were digital Arabic numerals (6 and 9) whose height and width were 1.0° and 0.75°, respectively. The stimuli were composed of seven line elements, and each line was 5.6 arcmin thick. These stimuli were named digits “6” and “9.” We also mirror-inverted the digits “6” and “9,” using them as the target. These stimuli were named inverted “6” and “9.” Therefore, four target types were used: digits “6” and “9” and inverted “6” and “9.” Also, we used the digit “8” as the distractor (i.e., nontarget stimulus, see Procedure section for details) and the flanker, meaning that the distractor and flanker had no gap. The local components of the targets were the same, and the stimuli differed only in gap position, that is, the position where components were omitted from digit “8.” The targets were displayed together with one flanker only.

### Stimulus Presentations

A fixation cross was displayed at the center of the screen throughout the experiment, and stimuli were presented on the right side of the screen, that is, were always shown in the right visual field. The target was presented at 10° right of the fixation cross without vertical jitter, and the distractor was shown at the same location as the target. Four diagonal bars, with a length of 21 arcmin, were presented around the target position as the location cue throughout the experiment. The center-to-center distance between the target and flanker varied across seven levels: 0.94°, 1.09°, 1.41°, 2.03°, 3.28°, 4.53°, and 5.78°. The flanker was presented simultaneously with the target or distractor, and its position was fixed in one trial and varied between conditions.

**Figure 1.**
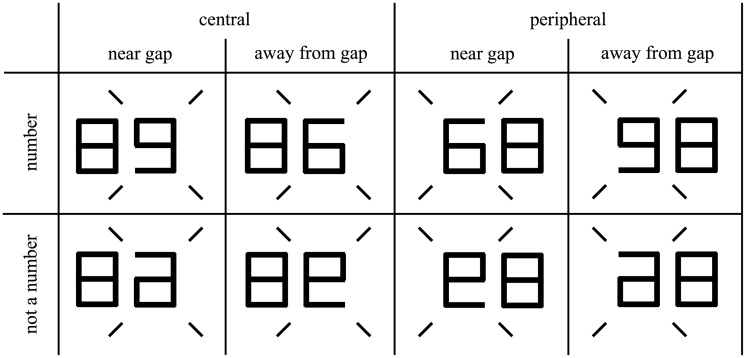
Experimental conditions summary. Three of the four factors (i.e., *gap*, *flanker location*, and *semantic category*) are depicted. The fourth factor is *center-to-center distance between the target and flanker*. Four diagonal bars were presented throughout the experiment as a cue of the target location.

We used a factorial design with four factors (Figure 1): *flanker location* (central or peripheral), *relative position of the target’s gap to the nearest flanker edge* (near-gap or away-from-gap), *target semantic category* (number or not-a-number), and c*enter-to-center distance between the target and flanker*. In total, there were 56 conditions (2 × 2 × 2 × 7).

### Procedure

**Figure 2.**
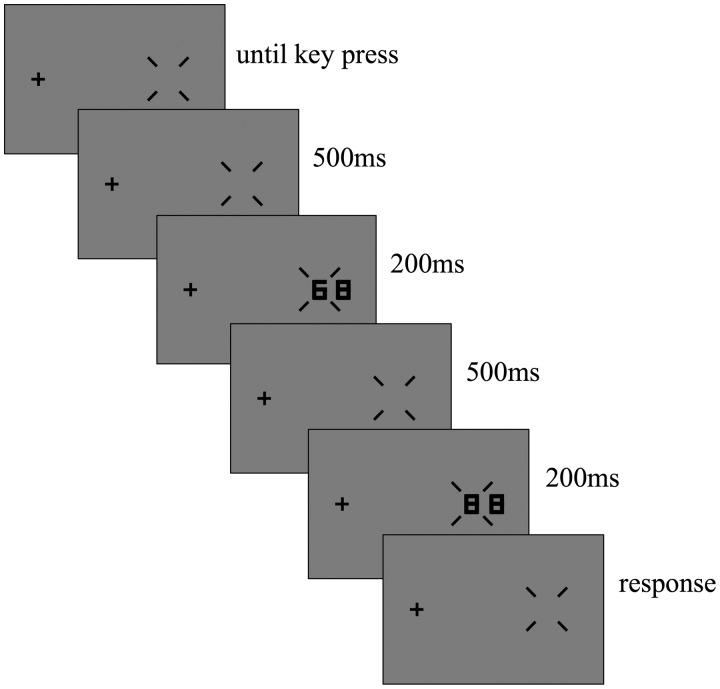
Schematic description of the procedure. When observers pressed the space key, a blank screen appeared for 500 ms, after which the first and second stimuli were presented for 200 ms with 500-ms interstimulus interval. Then, only the fixation cross and diagonal bars were presented on the gray background. The observers were asked to indicate which stimulus interval had contained the target.

The experiment was conducted with a two-interval temporal forced-choice paradigm (Figure 2). At the start of each block, the fixation cross and cueing bars were presented; these stimuli were constantly shown throughout the experiment. When the observers pressed the space key, a middle tone beep sound (600 Hz) informed them that the trial sequence had started. After 500 ms, the first stimulus was presented for 200 ms, and with an interstimulus interval of 500 ms, the second stimulus was shown for 200 ms. Subsequently, only the fixation cross and cueing bars were displayed. The same flanker was shown in both stimulus intervals; however, the target was presented in only one, randomly chosen interval. The other interval contained the distractor (i.e., the digit “8”). By pressing the appropriate keys, the observers could indicate which interval contained the target. If they chose the first interval, they pressed the “f” key, and pressed the “j” key for the second one. A high tone beep sound (800 Hz) gave auditory feedback for correct answers, while a low tone beep (400 Hz) for incorrect ones. All the 56 conditions were shown in each block, in random order, and each condition was shown twice (resulting in 112 presentations per block). Ten blocks were conducted for each observer, with each condition repeating 20 times. The correct rate for each condition was calculated. Before the experiment, the observers took part in practice sessions, during which the flanker was not presented.

### Data Analysis

**Figure 3.**
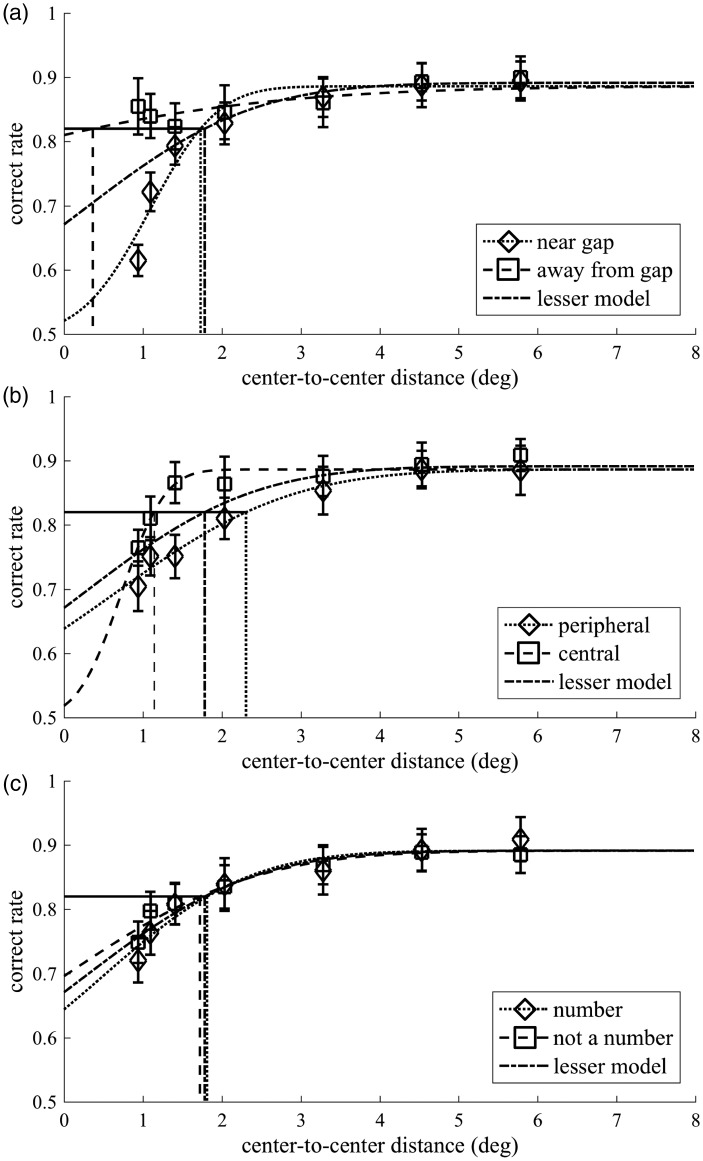
Psychometric functions. The correct rate averaged across observers is plotted as a function of center-to-center distance with square or diamond markers. Error bars indicate the standard error. For a psychometric function, we used a cumulative normal distribution function. The dashed and dotted lines indicate the respective psychometric function for (a) the factor of gap, (b) the factor of flanker location, and (c) the factor of semantic category. The dash-dotted lines show the respective lesser model. The solid horizontal and dashed or dotted or dash-dotted vertical lines mark the correct rate of 0.82 and critical distance for each psychometric function.

At first, to focus on the effects of three factors (i.e., gap, flanker location, and semantic category), we plotted the mean correct rates for each factor between observers as a function of center-to-center distance (Figure 3). For a psychometric function, we fitted a cumulative normal distribution function to the observed data using a maximum-likelihood method with the Palamedes Toolbox extensions for MATLAB (Prins & Kingdom, 2018). There were four parameters: α (which determines the psychometric function’s location on the abscissa, in units of deg), β (which is related to the psychometric function’s slope, or rate of change, in units of 1/deg), γ (which indicates the lower asymptote), and λ (which indicates the upper asymptote). In the cumulative normal distribution function, α corresponds to the mean (μ), 1/β corresponds to the variance (σ), γ corresponds to the guess rate, and 1-λ corresponds to the lapse rate. Among these, γ was always fixed at 0.5 because we used a two-alternative forced-choice paradigm. Subsequently, we compared two models, a fuller and a lesser model (following Prins & Kingdom, 2018). In the fuller model, two separate psychometric functions were fitted to each condition of each factor separately, whereas one common psychometric function was fitted to both conditions of each factor in the lesser model. More specifically, in the fuller model, α and β were free and different between conditions, while λ was free but the same between conditions. In the lesser model, α, β, and λ were free but the same between conditions. We calculated a transformed likelihood ratio (TLR) and tested whether the fuller model was significantly better than the lesser model in explaining the observed data by using an empirical sampling distribution based on 1,000 Monte Carlo simulations. Finally, we compared the better model, the fuller or lesser model, with a saturated model to perform a goodness-of-fit test using a likelihood ratio test by calculating a deviance (*D*) and using an empirical sampling distribution based on 1,000 Monte Carlo simulations.

## Results

The three psychometric function parameters, α, β, and λ were as defined earlier. In addition, we defined a critical distance (in units of deg) of each psychometric function. Critical distances are usually defined as the point in the middle of the psychometric function, that is, α. However, we operationally defined critical distance (center-to-center) as the point on the respective psychometric function where the correct rate reaches 0.82, which is higher than the point in the middle of the psychometric function (i.e., a critical distance will be larger than α). In the present study, α is ill-defined in several conditions, such that, for example, it gets a negative value, due to missing data at sufficiently small distances. Indeed, to describe and compare the characteristics of each psychometric function with meaningful values, we used critical distance as defined earlier, with the criterion chosen at 0.82 proportion correct because it is the point in the middle between 0.75 (the point of inflection) and 0.89 (the upper asymptote of psychometric functions). A summary of the parameter values in the various conditions is shown in Table 1.

**Table 1.** Summary of Parameters of Psychometric Functions.

	Gap			Flanker location			Semantic category		
	Near-gap	Away-from- gap	Lesser model	Peripheral	Central	Lesser model	Number	Not-a- number	Lesser model
α	1.09	−3.21	0.27	0.64	0.73	0.27	0.49	0.00	0.27
β	1.48	0.27	0.60	0.57	2.30	0.60	0.69	0.53	0.60
λ	0.11	0.11	0.11	0.11	0.11	0.11	0.11	0.11	0.11
Critical distance	1.73	0.36	1.78	2.31	1.14	1.78	1.81	1.72	1.78
Deviance	8.48			3.49			4.79		

### Gap

Figure 3(a) shows the psychometric functions for the near-gap and away-from-gap conditions as well as the lesser model for the gap factor. The parameters for the near-gap condition are as follows: α = 1.09, β = 1.48, and λ = 0.11. The parameters for the away-from-gap condition are as follows: α = −3.21, β = 0.27, and λ = 0.11. Estimated critical distance was 1.73° and 0.36° for the near-gap and away-from-gap condition, respectively. The parameters for the lesser model are as follows: α = 0.27, β = 0.60, and λ = 0.11. Estimated critical distance was 1.78° for the lesser model. The model comparison showed that the fuller model was significantly better than the lesser model for fitting the observed data (TLR = 37.8, *p* = .001). The goodness-of-fit test indicates that the fuller model is a reasonable fit to the observed data (*D* = 8.48, *p* = .866).

### Flanker Location

Figure 3(b) shows the psychometric functions for the peripheral and central conditions as well as the lesser model for the flanker location factor. The parameters for the peripheral condition are as follows: α = 0.64, β = 0.57, and λ = 0.11. The parameters for the central condition are as follows: α = 0.73, β = 2.30, and λ = 0.11. Estimated critical distance was 2.31° and 1.14° for the peripheral and central condition, respectively. The parameters for the lesser model are as follows: α = 0.27, β = 0.60, and λ = 0.11. Estimated critical distance was 1.78° for the lesser model. The model comparison showed that the fuller model was significantly better than the lesser model for fitting the observed data (TLR = 16.6, *p* = .003). The goodness-of-fit test indicates that the fuller model is a reasonable fit to the observed data (*D* = 3.49, *p* = .998).

### Semantic Category

Figure 3(c) shows the psychometric functions for the number and not-a-number conditions as well as the lesser model for the semantic category factor. The parameters for the number condition are as follows: α = 0.49, β = 0.69, and λ = 0.11. The parameters for the not-a-number condition are as follows: α = 0.00, β = 0.53, and λ = 0.11. Estimated critical distance was 1.81° and 1.72° for the number and not-a-number condition, respectively. The parameters for the lesser model are as follows: α = 0.27, β = 0.60, and λ = 0.11. Estimated critical distance was 1.78° for the lesser model. The model comparison showed that the fuller model and the lesser model were not significantly different (TLR = 0.907, *p* = .672); therefore, we adopted the lesser model. The goodness-of-fit test indicates that the lesser model is a reasonable fit to the observed data (*D* = 4.79, *p* = .941).

## Discussion

This study introduced a novel gap detection paradigm, examining within a single task how multiple types of proximity affect the degree of crowding. We assumed three different types of proximity; the relative position between the target gap and nearest flanker edge (near-gap or away-from-gap), the flanker location compared with the target location (central or peripheral), and the semantic category of the target (number or not-a-number). Effects of the former two types of proximity were observed, whereas no effect of the semantic category was found.

The model comparison for the gap factor revealed that the psychometric functions for the near-gap and away-from-gap conditions were significantly different. The critical distance in the away-from-gap condition was smaller than that in the near-gap condition. These results showed that the proximity between the target gap and nearest flanker edge affected the degree of crowding. Figure 3(a) shows that the difference between conditions was particularly evident when the center-to-center distance was short, and the correct rate in the away-from-gap condition remained high even when center-to-center distance was shortest. Thus, at equal center-to-center distances, the correct rate differs widely between the near-gap condition and away-from-gap condition, confirming the importance of edge-to-edge distance between target gap and flanker (Bouma, 1970; Rosen, Chakravarthi, & Pelli, 2014). Due to the edge-to-edge local interaction, crowding may be related to contour integration at an early stage of visual processing (Flom, Weymouth, & Kahnemann, 1963; May & Hess, 2007), wherein local components are connected to represent smooth contours within a small association field (Field, Hayes, & Hess, 1993).

The model comparison for the flanker location factor showed that the psychometric functions for the central and peripheral conditions were significantly different. The critical distance was larger in the peripheral condition than in the central condition. These results mean that the flanker presented more peripherally than the target impaired the task more strongly than the flanker presented more centrally than the target did, indicating a central-peripheral asymmetry of crowding (Bex et al., 2003; Bouma, 1973; Chastain, 1983; Dayan & Solomon, 2010; Estes et al., 1976; Estes & Wolford, 1971; Haslerud & Clark, 1957; Krumhansl, 1977; Mackworth, 1965; Petrov & Popple, 2007; Petrov et al., 2007; Toet & Levi, 1992; but see also Chastain, 1982; Strasburger & Malania, 2013; see Strasburger, 2018, for a recent review). Considering the possible relationship between central-peripheral asymmetry and the cortical magnification factor (Motter & Simoni, 2007; Nandy & Tjan, 2012), the larger critical distance with the peripheral condition might partially be explained in terms of the smaller representation of the target-flanker distance in the visual cortex. It should be noted, however, that there is the refutation by other previous studies that the cortical magnification factor cannot fully explain the central-peripheral asymmetry (Petrov & Meleshkevich, 2011; Petrov et al., 2007).

The model comparison for the semantic category factor showed that one common psychometric function was sufficient to explain the observed results in both the number and not-a-number conditions. Indeed, the results we obtained did not indicate an effect of the similarity of the semantic category between the target and flanker on crowding. This is inconsistent with previous studies that have shown a category similarity effect (Huckauf et al., 1999; Reuther &Chakravarthi, 2014; but see also Yu et al., 2012). This inconsistency might be due to the difference in task requirements between previous studies and this study. Studies on category effect asked observers to answer letter identity, such that the presented target was A, B, or X, making observers attend to whole parts of the target and its meaning. In contrast, this study instructed observers to detect target gaps, directing the focus of the observers onto detailed, local features of the target. Differences in a task set are known to affect performance despite the physical display remaining the same between conditions (e.g., Osugi, Hayashi, & Murakami, 2016). Thus, this difference might cause seemingly discrepant results. Therefore, attention to letter identity—a global characteristic of the target—might be necessary for the influence of the semantic category on crowding, but which did not apply to this study.

The psychometric function analysis has revealed limitations of the present study, or perhaps, limitations of the paradigm. In the present study, α was ill-defined in several conditions, such that it got a negative value (Table 1). Similarly, the psychometric function’s slope (i.e., β) was too shallow in some conditions compared with the ordinary psychometric function’s slope (e.g., Rosen et al., 2014). That should be because data at sufficiently small center-to-center distances were not acquired, indicating the limitation of the present study. This issue may be addressed by using smaller target-flanker distances, but it is even possible that data at sufficiently small flanker distances could not be acquired due to the design of the stimuli. In the present paradigm, only a single flanker was used, which may be insufficient to cause enough strong crowding. To collect data at sufficiently small separations, one possible approach is adding flankers above and below the target because more flankers can cause stronger crowding (e.g., Pelli et al., 2004). If these weaknesses are sufficiently addressed, the task used in this study, which enabled the measurement of different types of proximity within a single task, can become a new, useful tool for future research on crowding.
